# Subclinical Thyroid Dysfunction and Cognitive Decline in Old Age

**DOI:** 10.1371/journal.pone.0059199

**Published:** 2013-03-12

**Authors:** Liselotte W. Wijsman, Anton J. M. de Craen, Stella Trompet, Jacobijn Gussekloo, David J. Stott, Nicolas Rodondi, Paul Welsh, J. Wouter Jukema, Rudi G. J. Westendorp, Simon P. Mooijaart

**Affiliations:** 1 Department of Gerontology and Geriatrics, Leiden University Medical Center, Leiden, The Netherlands; 2 Department of Cardiology, Leiden University Medical Center, Leiden, The Netherlands; 3 Department of Public Health and Primary Care, Leiden University Medical Center, Leiden, The Netherlands; 4 Academic Section of Geriatric Medicine, Faculty of Medicine, University of Glasgow, Glasgow, Scotland; 5 Department of General Internal Medicine, Inselhospital, University of Bern, Bern, Switzerland; 6 British Heart Foundation Glasgow Cardiovascular Research Centre, University of Glasgow, Glasgow, Scotland; 7 Institute for Evidence-Based Medicine in Old Age | IEMO, Leiden, The Netherlands; IPATIMUP/Faculty of Medicine of the University of Porto, Portugal

## Abstract

**Background:**

Subclinical thyroid dysfunction has been implicated as a risk factor for cognitive decline in old age, but results are inconsistent. We investigated the association between subclinical thyroid dysfunction and cognitive decline in the PROspective Study of Pravastatin in the Elderly at Risk (PROSPER).

**Methods:**

Prospective longitudinal study of men and women aged 70–82 years with pre-existing vascular disease or more than one risk factor to develop this condition (N = 5,154). Participants taking antithyroid medications, thyroid hormone supplementation and/or amiodarone were excluded. Thyroid function was measured at baseline: subclinical hyper- and hypothyroidism were defined as thyroid stimulating hormones (TSH) <0.45 mU/L or >4.50 mU/L respectively, with normal levels of free thyroxine (FT4). Cognitive performance was tested at baseline and at four subsequent time points during a mean follow-up of 3 years, using five neuropsychological performance tests.

**Results:**

Subclinical hyperthyroidism and hypothyroidism were found in 65 and 161 participants, respectively. We found no consistent association of subclinical hyper- or hypothyroidism with altered cognitive performance compared to euthyroid participants on the individual cognitive tests. Similarly, there was no association with rate of cognitive decline during follow-up.

**Conclusion:**

We found no consistent evidence that subclinical hyper- or hypothyroidism contribute to cognitive impairment or decline in old age. Although our data are not in support of treatment of subclinical thyroid dysfunction to prevent cognitive dysfunction in later life, only large randomized controlled trials can provide definitive evidence.

## Introduction

Subclinical thyroid dysfunction, defined biochemically as a normal serum level of free thyroxine (FT4) in the presence of a high or low level of thyroid stimulating hormone (TSH), are a common finding among the elderly population with a prevalence of up to 20% [1]. Both subclinical hyper- and hypothyroidism have been implicated as risk factors for cognitive decline, although the literature regarding these associations shows inconsistent results [2]–[8].

A candidate mechanism to explain a possible association of subclinical thyroid dysfunction with cognitive decline is cardiovascular disease. Cardiovascular disease and its risk factors are important contributors to cognitive decline in later life [9]. Hypothyroidism is associated with hypertension [10], [11], and higher levels of total cholesterol and low-density lipoprotein (LDL) cholesterol [12]–[14], both of which increase the risk of atherosclerosis [15], [16]. A recent analysis of 55,287 individual participant data from 11 prospective cohort studies showed that the subclinical stage of hypothyroidism is associated with an increased risk of coronary heart disease events and mortality [17]. Furthermore, it is well established that subclinical hyperthyroidism is related to an increased risk of atrial fibrillation [15], [18], [19], which is an important risk factor for stroke. Hence, both subclinical hyperthyroidism and hypothyroidism are associated with cardiovascular disease and could therefore be important contributors to cognitive decline. Inconsistent results of previous studies on the association of subclinical thyroid dysfunction and cognition could be explained by small numbers of participants, low burden of cardiovascular disease and scarcity of longitudinal data.

In the present study, we examined the association of subclinical thyroid dysfunction and cognitive decline in the PROspective Study of Pravastatin in the Elderly at Risk (PROSPER), which consisted of 5,154 participants aged 70–82 years with pre-existing vascular disease or risk factors thereof.

## Methods

### Subjects

Participants were part of PROSPER, a multicentre, randomized placebo-controlled trial [20]. PROSPER was designed to test the hypothesis whether treatment with pravastatin reduces the risk of subsequent major vascular events in a cohort of elderly men and women with pre-existing vascular disease or at risk of developing this condition [20]. Briefly, 5,804 participants aged 70 to 82 years with pre-existing vascular disease or who had more than one risk factor to develop this condition (defined as hypertension, cigarette smoking or diabetes mellitus), were randomized to receive treatment with 40 mg pravastatin per day or matching placebo. After a 3 to 5-year intervention period, assessments were made on the influence of this therapy on major vascular events (a combination of coronary heart disease death, nonfatal myocardial infarction, and fatal and nonfatal stroke). No effect was found of pravastatin treatment on cognitive performance during follow-up, nor was there an effect of pravastatin on TSH during follow-up [21]. The study was approved by the institutional ethics review boards of centres of Cork University (Ireland), Glasgow University (Scotland) and Leiden University Medical Center (the Netherlands) and all participants gave written informed consent.

### Thyroid function

Because of the distorting effect of antithyroid medications (Carbimazole, Methimazole and Propylthiouracil), thyroid hormone supplementation (Levothyroxine) and amiodarone on thyroid function and on cardiovascular disease [22], participants using any of these medications at baseline were excluded (n = 6, n = 159 and n = 20 respectively). Blood samples in PROSPER were collected at baseline. Thyroid function was determined by state-of-the-art serum immunoassays for TSH (third generation assays with functional sensitivity of 0.05 mIU/l or less) and for FT4 in respective laboratory centers (Cork, Ireland; Glasgow, Scotland; and Leiden, the Netherlands). Inter- and intra-assay coefficients of variation were less than 5% for both TSH and FT4. To account for the differences of laboratory assays, we used a narrow FT4 reference range; values of 12 pmol/L to 18 pmol/L were considered normal [23]. For TSH, we used a reference range derived from relevant literature; values of 0.45 mU/L to 4.50 mU/L were considered as normal [1], [17]. Subclinical thyroid dysfunction is defined as a normal serum level of free thyroxine in the presence of a low or high level of thyroid stimulating hormone. We defined euthyroidism as a normal serum level of TSH.

To assess the risks associated with persistent subclinical thyroid dysfunction [24], TSH and FT4 measurements were repeated at 6 months in archived serum samples, which were stored at −70°C in the Glasgow University laboratory. TSH and FT4 were measured using the same electrochemiluminescence immunodetection method on a Roche Elecsys 2010 (Burgess Hill; UK). The limit of detection of TSH was <0.005 uIU/ml; for FT4 this was 0.3 pmol/L with a reference range of 12 pmol/L to 22 pmol/L [23].

### Cognitive performance

Cognitive performance was tested at baseline, after 9, 18, 30 months and at the end of the study. The time point of this last measurement varied between 36 and 48 months; therefore, we performed the analyses with their individually varying time-point, but report the results for the mean of these time points (at 42 months).

The Mini-Mental-State-Examination (MMSE) was used to screen for global cognitive dysfunction; participants with a baseline MMSE-score below 24 points were excluded from PROSPER [20]. Furthermore, four neuropsychological performance tests were used to measure executive function and memory [25]. The Stroop-Colour-Word-Test was used to test selective attention and reaction time of the participants. The participants were asked to read a colour name which was displayed in a colour different from the colour it actually names. The outcome parameter was the total number of seconds to complete the test; a higher score therefore indicates worse performance. General cognitive speed was tested by the Letter-Digit Coding Test. The participants had to match certain digits with letters according to a provided key. The outcome variable was the total number of correct entries in 60 seconds, and therefore higher scores represents better performance. The Picture-Word Learning Test was used as a verbal learning test of long-term memory. Fifteen pictures were presented at the participants, and they were asked to recall as many pictures as possible in three trials. After 20 minutes they were asked to repeat the test to measure their delayed recall. The outcome parameter is the accumulated number of correct recalled pictures, immediate and after 20 minutes. Higher scores thus indicate better performance.

### Statistical methods

Participants with subclinical hyperthyroidism and subclinical hypothyroidism were compared to participants with euthyroidism at baseline by calculating means and percentages of baseline characteristics. To investigate the cross-sectional association between subclinical thyroid function and cognitive performance, we performed linear regression analyses. Adjustments were made for potential confounders; sex, age, education, country, apo E genotype, and where appropriate for version of cognitive test. The effect of thyroid function on cognitive performance during follow-up was investigated by linear mixed models for repeated measurements. The models included baseline thyroid function (subclinical hyperthyroidism, euthyroidism and subclinical hypothyroidism), time (in years) and the interaction term between time and thyroid function. The dependent variable was the series of repeated measurement of cognitive performance during follow-up. The estimated values for thyroid function indicates the cross-sectional difference in cognition between the two subclinical groups, as compared to the euthyroid group. The change in cognition per year during the follow-up period is represented by the estimated value for annual change over time. The estimated value for the interaction between time and thyroid function represents the additional change in cognition per year in participants with subclinical hyper- or hypothyroidism compared to euthyroidism. A significant difference in this term would indicate that cognitive decline over the mean follow-up period of 3.2 years differed between the subclinical thyroid groups and euthyroidism. Adjustments were made for sex, age, education, country, study treatment, apo E genotype and where appropriate for version of cognitive test.

Moreover, to further explore the association between subclinical thyroid dysfunction and cognitive performance, we performed several sensitivity analyses in which we 1) used a wider FT4 reference range, with 10.3 pmol/L to 25.7 pmol/L considered normal according to relevant literature values [1]; 2) excluded all participants who started thyroid hormone therapy and/or antithyroid medications and/or amiodarone during follow-up; 3) excluded all participants who developed heart failure during follow-up; and 4) only included participants with persistent subclinical hyper- or hypothyroidism or persistent euthyroidism, defined as subclinical hyper- or hypothyroidism or euthyroidism at both baseline and six months. Additionally, we investigated whether an association existed between subclinical thyroid status and cognitive function and decline, which was specific for a lower or higher global cognitive function (i.e. below or above the median MMSE score of 27 points) at baseline and which may have been obscured by analyzing the cohort as a whole. We did similar analyses for participants with and without pre-existing vascular disease or risk factors (current smoking, history of diabetes and/or history of hypertension) thereof; with an age of leaving school below or above the median value of 14 years; and with a very low (<0.1 mU/L) or very high TSH-value (>10 mU/L). P-values less than 0.05 were considered statistically significant. All analyses were performed with SPSS (version 17.0, PASW Statistics Inc, Chicago, III).

## Results

**Figure 1:**
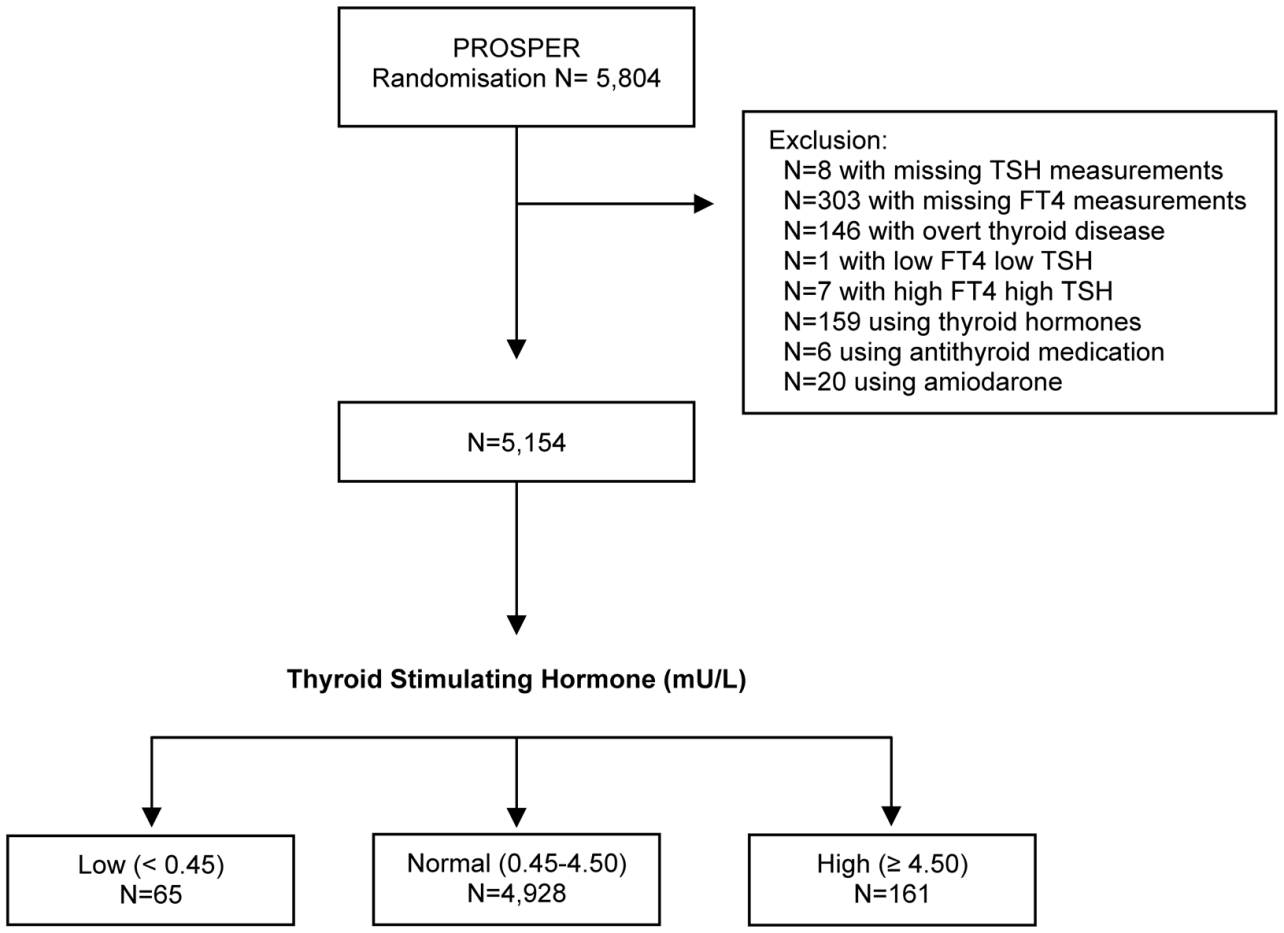
Flowchart of study participants. Abbreviations: TSH, Thyroid Stimulating Hormone; FT4, free thyroxine.

Figure 1 shows the flowchart of study participants. From all participants of PROSPER (n = 5,804), a number of 650 were excluded, resulting in 5,154 participants for the present analyses. We categorized these participants into three different thyroid status groups. A total of 4,928 participants were euthyroid. Subclinical hyperthyroidism was found in 65 of the participants. Subclinical hypothyroidism was found in 161 of the participants. Treatment with pravastatin was evenly distributed among the three groups (data not shown).

Table 1 shows the baseline characteristics of the study participants per thyroid status group. In euthyroid participants, gender was almost evenly distributed (48.6% females), while in both subclinical groups females were overrepresented (73.8% and 64.0%, both p-values <0.01). There was no significant age difference between the three groups. All three groups had a similar score on Barthel test and on Instrumental Activities of Daily Living scale.

**Table 1:** Baseline characteristics of study participants grouped by thyroid status.

	Thyroid status			p-value for difference	
	Subclinical hyperthyroidism N = 65	Euthyroidism N = 4,928	Subclinical hypothyroidism N = 161	Subclinical hyperthyroidism vs. euthyroidism	Subclinical hypothyroidism vs. euthyroidism
Demographics					
Female, n (%)	48 (73.8%)	2,394 (48.6%)	103 (64.0%)	<0.001	<0.001
Age (years), mean (se)	75.33 (0.38)	75.28 (0.05)	75.63 (0.25)	0.90	0.20
Education (years), mean (se)	15.29 (0.27)	15.14 (0.03)	15.17 (0.17)	0.56	0.89
Barthel score, mean (se)*	19.63 (0.09)	19.78 (0.01)	19.86 (0.06)	0.08	0.18
IADL score, mean (se) *	13.59 (0.12)	13.65 (0.01)	13.64 (0.08)	0.62	0.95
Biometrics, mean (se)*					
Weight (kg)	71.73 (1.51)	73.47 (0.17)	74.76 (0.96)	0.25	0.19
Height (cm)	166.60 (0.83)	165.43 (0.10)	166.58 (0.52)	0.15	0.03
BMI (kg/m^2^)	25.69 (0.51)	26.81 (0.06)	26.88 (0.33)	0.03	0.83
SBP (mmHg)	153.79 (2.70)	154.60 (0.31)	156.77 (0.72)	0.77	0.21
DBP (mmHg)	82.60 (1.42)	83.86 (0.16)	84.50 (0.90)	0.38	0.49
Risk factors, n (%)					
Current smoker	12 (18.5%)	1,367 (27.7%)	36 (22.4%)	0.10	0.13
History of diabetes	10 (15.4%)	525 (10.7%)	23 (14.3%)	0.22	0.14
History of hypertension	38 (58.5%)	3,008 (61.0%)	112 (69.6%)	0.67	0.03
History of vascular disease	25 (40.0%)	2177 (44.2%)	67 (41.6%)	0.50	0.52
History of TIA/stroke	6 (9.2%)	552 (11.2%)	20 (12.4%)	0.62	0.63
Lipid profile, mean (se)*					
HDL cholesterol (mmol/L)	1.24 (0.04)	1.28 (0.01)	1.27 (0.03)	0.32	0.62
LDL cholesterol (mmol/L)	3.82 (0.10)	3.78 (0.01)	3.86 (0.06)	0.69	0.20
Total cholesterol (mmol/L)	5.70 (0.11)	5.67 (0.01)	5.77 (0.07)	0.80	0.14
Triglycerids (mmol/L)	1.57 (0.09)	1.54 (0.01)	1.56 (0.05)	0.74	0.78

Abbreviations: n, number; se, standard error; IADL, Instrumental Activities of Daily Living; BMI, body mass index; SBP, systolic blood pressure; DBP, diastolic blood pressure; TIA, transient ischemic attack; HDL, high density cholesterol lipoprotein; LDL, low density cholesterol lipoprotein.

* =  adjusted for sex and age at baseline, assessed by linear regression.

In line with expectation, participants with subclinical hyperthyroidism had a lower body mass index compared to the euthyroid group (mean; 25.69 vs. 26.81, p = 0.03). There was no significant difference in systolic and diastolic blood pressure at baseline between the groups. Subclinical hypothyroidism was associated with a higher prevalence of hypertension (69.6% vs. 61.0%, p = 0.03). There was no significant difference in prevalence of smokers, history of diabetes, vascular disease and/or TIA/stroke between the groups. All three groups had a similar lipid profile.

The association of thyroid status and cognitive performance at baseline is presented in table 2. There was no significant difference in Mini-Mental State Examination, Stroop test, Letter-Digit Coding test and delayed Picture-Word Learning test at baseline between participants with subclinical hyperthyroidism or hypothyroidism when compared to participants with euthyroidism (all p-values>0.05). Participants with subclinical hypothyroidism had a higher score on immediate Picture-Word Learning test, although not statistically significant (mean; 9.45 vs. 9.72, p = 0.06).

**Table 2:** Association of subclinical thyroid status and various cognitive performance tests at baseline.

	Thyroid status			p-value for difference	
Cognitive test	Subclinical hyperthyroidism N = 65	EuthyroidismN = 4,928	Subclinical hypothyroidism N = 161	Subclinical hyperthyroidism vs. euthyroidism	Subclinical hypothyroidism vs. euthyroidism
MMSE, score	28.04 (0.19)	28.04 (0.03)	27.87 (0.12)	0.97	0.15
Stroop, seconds	65.61 (3.07)	65.34 (0.46)	65.90 (2.02)	0.93	0.78
LDCT, digits coded	24.31 (0.86)	23.63 (0.13)	23.51 (0.54)	0.42	0.79
PLTi, pictures remembered	9.29 (0.23)	9.45 (0.03)	9.72 (0.14)	0.50	0.06
PLTd, pictures remembered	10.31 (0.32)	10.30 (0.05)	10.65 (0.20)	0.97	0.09

Abbreviations: MMSE, Mini-Mental State Examination; LDCT, Letter-Digit Coding Test; PLTi, Picture-Word Learning Test immediate; PLTd, Picture-Word Learning Test delayed. Cognitive tests were presented in mean (standard error). Associations were assessed by linear regression analyses, adjusted for sex, age, education, country, apo E genotype and test version where appropriate.

The longitudinal association between thyroid status and cognitive decline is shown in table 3. As expected, there was a significant annual decline among the total study population in each cognitive performance test (all p-values<0.001), with an exception of the MMSE test (p-value = 0.31). The Mini-Mental State Examination showed an additional increase in participants with subclinical hypothyroidism compared to participants with euthyroidism (estimate; 0.08, p = 0.03). There was no significant additional change per year during follow-up in Stroop test, Letter-Digit Coding test and immediate and delayed Picture-Word Learning test scores in participants with subclinical hyper- and hypothyroidism compared to participants with euthyroidism.

**Table 3:** Association of thyroid status with cognitive performance during follow-up.

	Annual change in cognitive test score		Additional change			
Cognitive test	All participants		Subclinical hyperthyroidism vs. euthyroidism		Subclinical hypothyroidism vs. euthyroidism	
	Est (se)	p-value	Est (se)	p-value	Est (se)	p-value
MMSE, score	0.01 (0.01)	0.31	−0.03 (0.06)	0.61	0.08 (0.04)	0.03
Stroop, seconds	0.68 (0.07)	<0.001	−0.48 (0.64)	0.45	0.70 (0.41)	0.09
LDCT, digits coded	−0.35 (0.02)	<0.001	−0.10 (0.14)	0.45	−0.02 (0.09)	0.85
PLTi, pictures remembered	−0.02 (0.01)	<0.001	−0.07 (0.06)	0.23	0.03 (0.04)	0.46
PLTd, pictures remembered	−0.07 (0.01)	<0.001	−0.13 (0.08)	0.13	0.00 (0.05)	0.94

Abbreviations: Est, estimates; se, standard error; MMSE, Mini-Mental State Examination; LDCT, Letter-Digit Coding Test; PLTi, Picture-Word Learning Test immediate; PLTd, Picture-Word Learning Test delayed. Estimates represent the additional change in various cognitive performance tests per year in different subclinical thyroid status. Adjusted for sex, age, education, country, treatment, apo E genotype and test version where appropriate.

Furthermore, sensitivity analyses in which we 1) used a wider FT4 reference range with normal FT4 values between 10.3 pmol/L and 25.7 pmol/L; 2) excluded all participants who started thyroid hormone therapy and/or antithyroid medications and/or amiodarone during follow-up (n = 138); 3) excluded all participants who developed heart failure during follow-up (n = 200); and 4) only included participants with persistent subclinical hyperthyroidism (n = 41) or hypothyroidism (n = 90) or persistent euthyroidism (n = 4447), did not show any differences of our results (data not shown). Additional analyses in subgroups (participants with a MMSE-score below or above the median MMSE score, with or without pre-existing vascular disease, with or without risk factors for vascular disease (smoking, hypertension and/or diabetes), with an age left school below or above the median value and participants with a very low or very high TSH-value) also did not change our findings (data not shown).

Thyroid status was not associated with an altered risk of stroke during follow-up (data not shown). However, the number of strokes during follow-up was small (n = 242) and therefore power was limited. Excluding these participants from the analyses did not materially change our results (data not shown).

## Discussion

In this large prospective cohort study, we found no association between subclinical thyroid dysfunction and cognitive performance at baseline and during follow-up among 5,154 participants aged 70–82 years old with vascular disease or at risk thereof. This is the largest follow-up study with repeated measurement of cognitive performance and repeated TSH/FT4 measurements in an elderly population, which adds further knowledge to previous cross-sectional studies and shorter longitudinal studies [2]–[8], [26], [27].

Clinical guidelines for various diseases, including dementia, recommend screening for subclinical thyroid disease in old age [28]–[30]. Although the condition has been implicated as a risk factor for cognitive decline, the clinical relevance of this finding still remains controversial [31]–[33]. Literature about the association between subclinical thyroid dysfunction and cognitive performance has shown conflicting results [2]–[8], [26], [27]. A few cross-sectional studies showed that subclinical hyperthyroidism was associated with lower cognitive performance, although the number of participants with subclinical hyperthyroidism was relatively small [2], [3]. In one large cross-sectional study of 5,865 participants with a mean age of 73.6 years, no association was found between subclinical thyroid dysfunction and anxiety, depression or cognitive impairment [7]. One longitudinal study with 1,843 nondemented participants with a mean age of 68.8 years demonstrated an association between subclinical hyperthyroidism and an increased risk of dementia and Alzheimer's disease [6]. Finally, the scarce randomized controlled trials concerning this association included few participants (n<100) and had relatively short follow-up (12 months) [26], [27]. Only one trial showed an improvement of thyroid hormone replacement on cognitive performance, although mean age in the study was low (62 years); and therefore not representative for the elderly [26].

There might be several explanations why we found no association of subclinical thyroid dysfunction with cognitive performance or decline. In the first place, it is well-established that participants with subclinical hypothyroidism may revert to euthyroidism within a short time [24]. The temporary change of TSH concentration in non-thyroidal illness could be of too short duration to cause permanent brain lesions. However, sensitivity analyses with only inclusion of participants with persistent subclinical hyper- or hypothyroidism or persistent euthyroidism yielded no differences of our results. Nevertheless, this could be explained by lack of power. Second, high levels of TSH could serve as a possible protective mechanism, thereby preventing cognitive decline in the elderly. This is supported by a recent study that found positive effects and better survival in participants with higher levels of TSH [4]. The speculated beneficial effect of subclinical hypothyroidism on cognition could therefore conceal the negative effects of vascular disease or its risk factors on cognitive performance in our study.

The finding that subclinical hypothyroidism is associated with increased risk of coronary heart disease [10], [16], [17], [23] could be a prominent candidate mechanism of how the condition could lead to cognitive decline. Since PROSPER only consist of participants suffering from coronary heart disease or at risk thereof, our study population would be highly suitable to detect such an association. However, most of the associations found in subclinical hypothyroidism concerned in particular participants with a TSH concentration of 10 mU/L or above [17]; and our data only had a small number of participants with this TSH concentration (n = 21). Furthermore, although we know that vascular risk factors have a detrimental impact on middle age; their negative association with vascular disease seems to attenuate in old age. For instance, high total cholesterol concentration is not a risk factor for vascular disease in elderly people, but instead, is associated with a lower mortality and increased longevity [34], [35], also in the population under study [35]. Additionally, high blood pressure values even associate with better survival in the elderly [36]. Taking all data together, we favor the interpretation that there is insufficient evidence of a relationship of subclinical thyroid dysfunction and cognitive decline in old age.

A strength of our study is the large sample size of 5,154 elderly participants, with an overall high prevalence of subclinical thyroid dysfunction. As we assessed change in cognitive performance with several sensitive cognitive tests [25], it is unlikely that we missed a clinically significant cognitive decline in this population. During the mean follow-up period of three years the cognitive test battery was repeated up to five times in each individual, resulting in a detailed and reliable measurement of cognitive function and decline. Furthermore, the preexistence of vascular disease or at least one risk factor to develop this condition made our study population highly appropriate to detect a possible association.

Among limitations, PROSPER only consisted of participants with a MMSE score of 24 points or higher, which may limit the generalizability of our results to elderly with cognitive dysfunction. Furthermore, it is likely that, as a result of selection on MMSE score, the power to detect an annual decline in cognition might be limited in our study. However, we did find a significant annual decline in cognitive function for Stroop test, Letter-Digit Coding test and immediate and delayed Picture-Word Learning tests in our study population. Additionally, the cognitive test battery we used has been proven to detect differences in annual cognitive decline among different groups in earlier studies [37], [38]. Nevertheless, our study showed no consistent difference in cognitive decline between participants with subclinical hyperthyroidism, euthyroidism and subclinical hypothyroidism. Finally, we may have excluded participants who had clinically significant cognitive deficits due to thyroid dysfunction at baseline. However, this limitation has likely resulted in a more homogeneous study population.

In conclusion, we found no association between subclinical thyroid dysfunction and cognitive performance at baseline and during three years of follow-up in a large group of elderly people with vascular disease or risk factors. Although our data are not in support of treatment of subclinical thyroid dysfunction to prevent cognitive dysfunction in later life, only large randomized controlled trials can provide definitive evidence.
